# Biological and Radiological Findings of Acquired Anterior Open Bite: A Case Report

**DOI:** 10.1155/crid/6670262

**Published:** 2025-11-14

**Authors:** Hanen Ben Khalifa, Raja Chebbi, Sayma Zegdene, Touhami Ben Alaya, Monia Dhidah

**Affiliations:** ^1^Department of Functional Exploration, Pain and Orofacial Dysfunction, University Dental Clinic, Monastir, Tunisia; ^2^Laboratory of Dento-Facial, Clinical and Biological Approach (ABCDF), Faculty of Dental Medicine, University of Monastir, Monastir, Tunisia; ^3^Department of Medical Imaging, University Dental Clinic, Monastir, Tunisia; ^4^Research Laboratory of Bioactive Natural Substances and Biotechnology (LR24ES14), Faculty of Dental Medicine, University of Monastir, Monastir, Tunisia

**Keywords:** bone resorption, case report, open bite, rheumatoid arthritis, temporomandibular joint

## Abstract

A 60-year-old female patient was referred to the Department of Functional Exploration, Pain, and Orofacial Dysfunction complaining of a pain in the preauricular regions and a recent apparition of an open bite impeding the masticatory function. In the general examination, the patient had a medical history of polyarthralgia with 7 years of evolution. She had no history of trauma in the maxillofacial region. Clinical examination revealed a restricted mouth opening and pain in the right and left temporomandibular joint (TMJ) areas. The palpation of this region revealed the presence of crepitations. A computed tomography (CT) scan of TMJ revealed a flattening of the condylar heads of the mandible. It also confirmed that the resorption of the two mandibular condyles is the origin of the open bite. After biological analysis, the diagnosis of TMJ disorder related to rheumatoid arthritis (RA) was made.

## 1. Introduction

Anterior open bite (OB) is a vertical malocclusion with multiple causes. It usually occurs at an early age and has either dental or skeletal origin. It may also appear when patients have certain parafunctional habits as thumb sucking or tongue thrusting [1].

Yet, when an OB appears at an old age in patient with no such habits but rather pain and restriction in mandibular movement, it can reveal a progressive destructive process in the temporomandibular joints (TMJs).

Joint destructions that lead to the appearance of an OB are usually part of a rapid destructive rheumatic disease such as rheumatoid arthritis (RA), juvenile RA, psoriatic arthritis, and some very rare connective tissue diseases rather than a typical secondary degenerative joint disease of the TMJ [2].

The treatment of TMJ rheumatic diseases is not consensual and aims above all to relieve pain, preserve the function, and prevent or delay complications in order to maintain the patients' quality of life.

The aim of this article is to describe the diagnostic and therapeutic approaches of an OB secondary to bone remodeling in the mandibular condyles in a 60-year-old female Tunisian patient in a chronic inflammatory rheumatic disease: the RA.

## 2. Case Report

A 60-year-old woman was referred by her treating dentist to the Department of Functional Exploration, Pain, and Orofacial Dysfunctions of the Dental Clinic of Monastir, Tunisia, with an OB and facial pain evolving for a year.

A history of pain in the preauricular regions, experienced as an ear pain, and a recent occurrence of an OB impeding the masticatory function were reported by the patient. These symptoms are aggravated by function. TMJ noises were also reported. She had no history of trauma or infection of the TMJ. The patient had a medical history of osteoporosis and polyarthralgia diagnosed by her family doctor 7 years ago and treated by nonsteroidal anti-inflammatory (NSAI) agents in addition to corticoids in the acute phases. These symptoms were noticed a year ago and have slowly worsened leading to a functional handicap.

**Figure 1:**
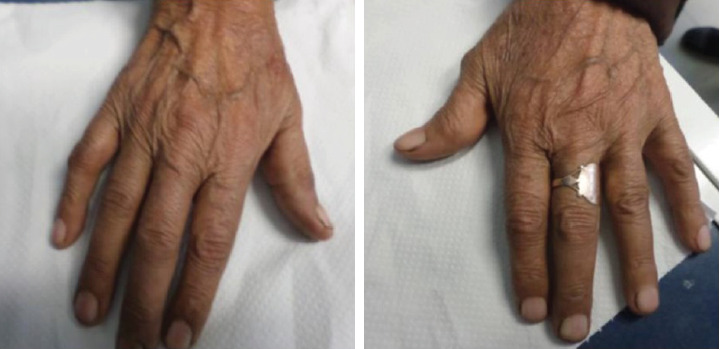
Boutonniere deformities of the interphalangeal joints affecting the patient fingers especially marked at the left thumb and right little finger.

The physical examination of the patient revealed a generalized polyarthralgia affecting her elbows, shoulders, knees, and wrists. Boutonniere deformities of the interphalangeal joints were especially marked at the left thumb and right little finger (Figure 1).

**Figure 2:**
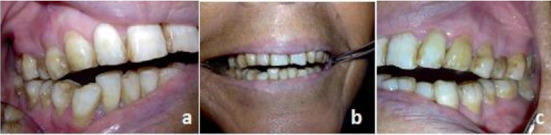
The open bite with no anterior contact to the second molar from both sides. (a) Right side view; (b) front view, and (c) left side view.

**Figure 3:**
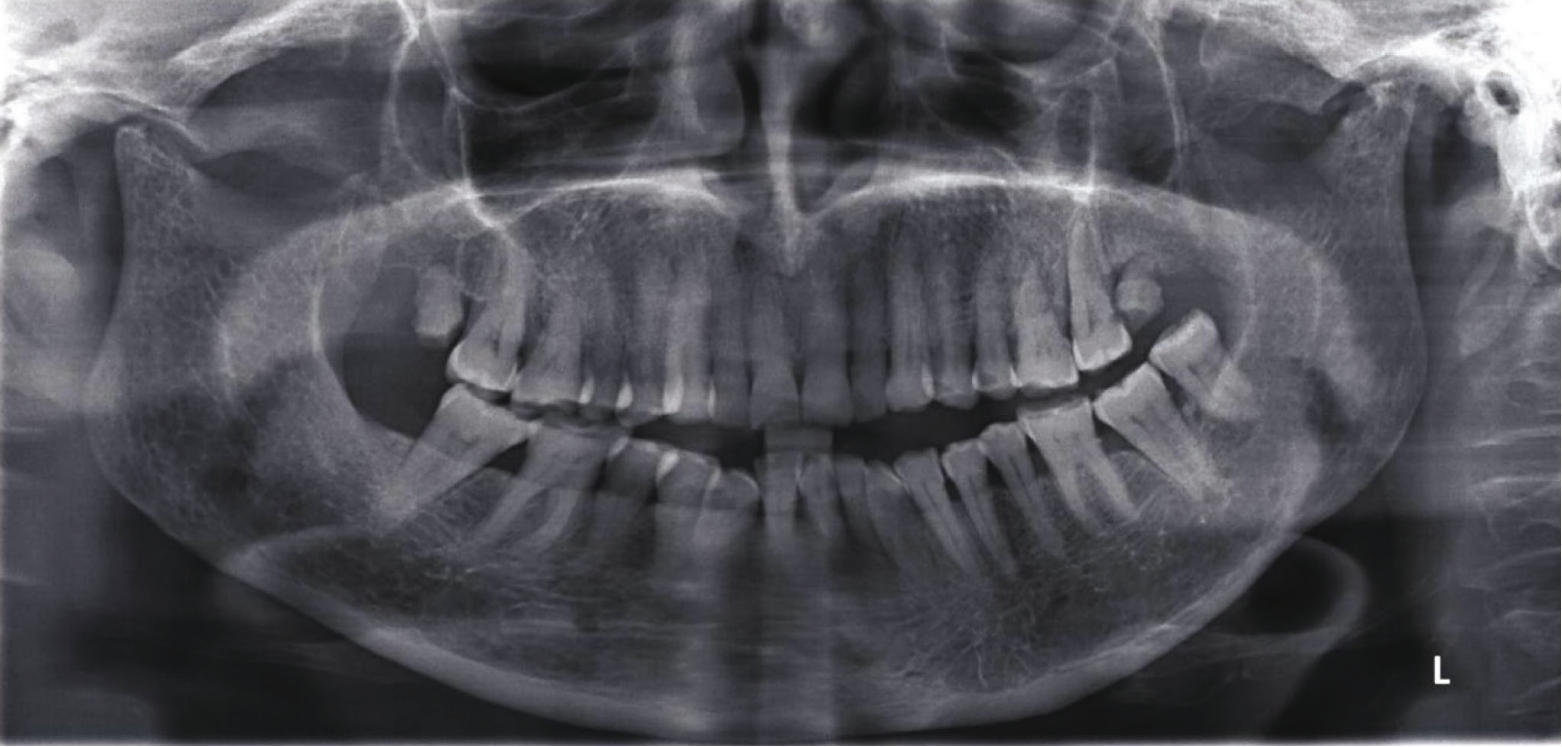
Orthopantomogram showing a bone lysis in both mandibular condyles, more advanced in the right one with irregular erosions and more pronounced in the left condyle.

At clinical examination, a bilateral stiffness in masticatory muscles with an articular pain in both TMJ regions was noticed. Crepitations were also found at TMJ auscultation. All mandibular movements (mouth opening, propulsion, and lateral excursion) were restricted with a bayoneted trajectory of the mouth opening. The occlusion examination revealed a wild OB with no anterior contact to the second molar from both sides (Figure 2). As a first step in the radiological exploration, a panoramic radiograph was performed. It depicted a bone lysis in both mandibular condyles more advanced in the right one with irregular erosions more pronounced in the left condyle. This bone loss could explain the anterior OB (Figure 3).

**Figure 4:**
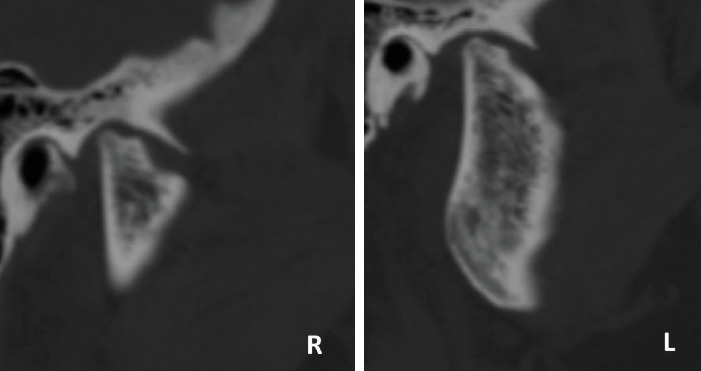
Para sagittal CT scan section at the right and left TMJ showing bone demineralization; a very advanced resorption of condylar process with a subtotal destruction of the condylar heads and loss of their normal radiological anatomy. R: right TMJ; L: left TMJ.

**Figure 5:**
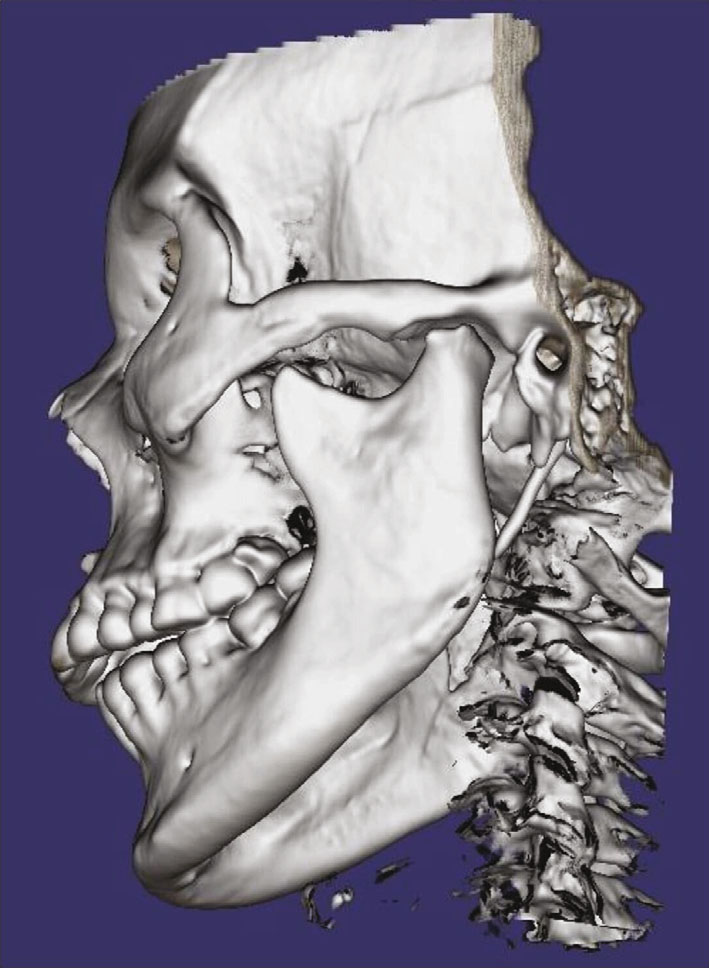
Three-dimensional reconstruction of the skull showing the resorption of the condyle, the shortening of the mandibular ramus and the anterior open bite.

In order to have more accurate images and to better assess bony changes, further evaluation was carried out by computed tomography scan (CT scan). It showed diffuse bone demineralization, more pronounced at the maxillary and mandibular bones, with no focal bone lesion or cortical lysis. There was a symmetrical and regular deformity with flattening of the condylar heads of the mandible, while the cortical bone and temporal slope of the TMJ were preserved (Figure 4). These findings confirmed that the resorption of the TMJ is the origin of the OB (Figure 5).

**Figure 6:**
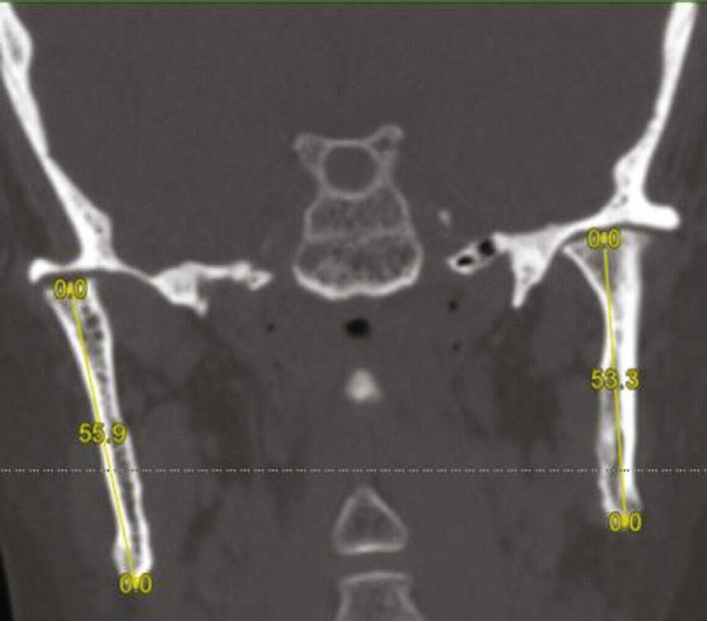
Coronal radiological section passing through the condylar process of the mandible showing the flattening of the condylar heads, the shortening of the mandibular rami (right ramus = 55.9 mm; left ramus = 53.3 mm) and the narrowing of joint space which is more pronounced in the left TMJ.

The coronal radiographic section of the condylar head showed a shortening of the mandibular rami which was slightly more pronounced in the left side (right ramus = 55.9 mm; left ramus = 53.3 mm) and a narrowing of the articular space bilaterally (more on the left) (Figure 6).

**Figure 7:**
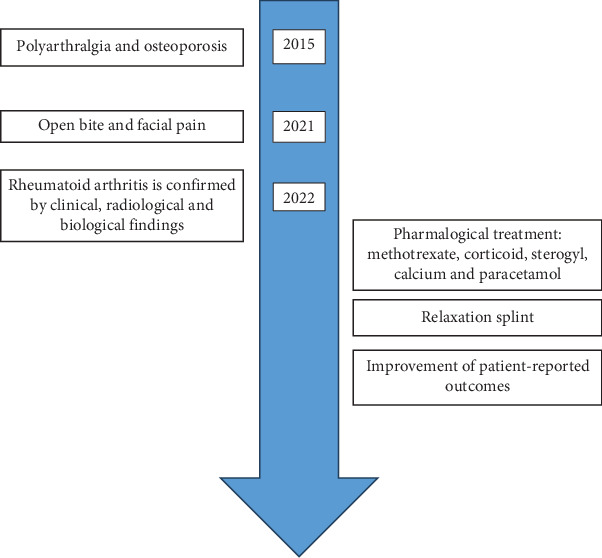
Timeline.

In front of these clinical and radiological data, an RA was suspected, and the patient was first referred to the rheumatology department where she was hospitalized urgently. After biological exploration, the diagnosis of RA was retained. The patient was put on methotrexate, corticoid, Sterogyl, calcium, and paracetamol. Then, she was referred to the maxillofacial surgery department for advice and possible treatment for reconstruction of the condylar heads. However, the general condition of the patient has contraindicated such an operation (Figure 7).

Therefore, a hard, flat surface relaxation splint restoring the maximum of contacts has been made. It was designed to reduce muscle tension and to alleviate TMJ symptoms. A notable improvement in patient-reported outcomes including reduced pain levels was observed.

## 3. Discussion

Resorption of the mandibular condyle has multifactorial origins. The clinical manifestations are essentially joint pain and occlusal disorders, depending on the rate of bone lysis. In some advanced cases, the resorption of the mandibular condyle may generate morphologic collapse of the TMJ causing shortening of the ramus, which results in progressive mandibular retrusion with anterior OB. This is called acquired OB associated with temporomandibular osteoarthritis (OA) [3].

In this case report, the progressive OB was the main concern of the patient. The clinical examination revealed joint crepitus and restricted mandibular movements that can lead the diagnosis to resorption of the mandibular condyle. The x-rays performed affirmed the global loss of volume in the condyle and, more particularly, the head.

A rheumatologic disease was suspected. After biological explorations, the diagnosis of RA was confirmed. The appearance of the OB attests the long-term evolution of the disease which was diagnosed at an advanced stage after severe joint destruction.

The prevalence of TMJ disorders in RA patients varies widely. Bracco et al. reported TMJ involvement in 53%–93% of patients [4], while Lin et al. reported radiological TMJ abnormalities in 74.5% of cases and functional abnormalities in 85.7% of them [5]. Ogus found affected TMJ in 61% of symptomatic patients [6].

Pain is considered the major factor that reduces the quality of life for patients with RA and the main reason for those patients to seek treatment. Yet other symptoms such as limitation of jaw function due to restriction of condylar translation and anterior opening of the bite due to articular cartilage and bone tissue destruction can also be very handicapping. That is why radiological examination seems to be useful and important for diagnosis and assessment of TMJ involvement. In fact, it makes it possible to reduce structural and functional damage by detecting bony changes at an early stage. Advanced imaging modalities such as CT scan or cone beam computed tomography (CBCT) are considered the best recommended imaging modalities for the degenerative diseases of TMJ as mild bony changes not demonstrated by conventional radiograph are clearly seen by these radiological techniques [7]. Degenerative changes of TMJ may also be seen in case of OA. However, RA and OA exhibit distinct radiological and clinical features. Radiologically, RA typically presents with erosive changes, such as bone erosion and joint space narrowing, often accompanied by soft tissue swelling and synovial proliferation, reflecting its inflammatory nature. In contrast, OA is characterized by degenerative changes, including osteophyte formation, subchondral sclerosis, and joint space narrowing without the extensive erosive changes seen in RA. Clinically, patients with RA may experience bilateral TMJ involvement, significant pain, and functional limitations due to inflammation. However, OA often presents with unilateral symptoms, crepitus, and pain that worsens with activity but improves with rest.

As clinical manifestations of the RA remain very diverse, the treatment of RA must be adapted not only to the stage of the damage but also to the characteristics of the patient. The new therapeutic concept of the RA advocates the establishment of a treatment in nonspecific arthritis before even existence of all the diagnostic criteria of RA, to associate several therapies and to establish close surveillance and a quick adjustment of the treatment if necessary [8].

Therefore, in addition to the pharmacological treatment, a splint therapy was also instated. A relaxation splint was made to ease the muscular pain and establish the maximum of dental contacts relieving the TMJ from the pressure.

## 4. Conclusion

Recent and rapid appearance of anterior OB can be indicative of a collapse of the mandibular condyle as part of a systemic disease. Therefore, we must push the interrogation and carry out complete clinical, radiological, and biological examinations in order to find out the cause of this osteoarthritic reshaping. Early diagnosis can lead to an appropriate treatment plan to stop the degenerative process and prevent much more serious complications that can affect patients' quality of life.

This case report attests the involvement of the TMJ in RA which can lead to severe occlusal alterations due to articular destruction and highlights the role of radiological exploration especially advanced imaging in diagnosing bony degenerative changes at an early stage to avoid such complications. Moreover, it witnesses the need for multidisciplinary healthcare in order to delay the degenerative process, abolish pain, and restore a normal manducatory function.

## Data Availability

The data supporting the findings of this study are available on request from the corresponding author. The data are not publicly available due to privacy or ethical restrictions.
